# Investigation of the Antiproliferative Properties of Natural Sesquiterpenes from *Artemisia asiatica* and *Onopordum acanthium* on HL-60 Cells *in Vitro*

**DOI:** 10.3390/ijms17020083

**Published:** 2016-02-17

**Authors:** Judit Molnár, Gábor J. Szebeni, Boglárka Csupor-Löffler, Zsuzsanna Hajdú, Thomas Szekeres, Philipp Saiko, Imre Ocsovszki, László G. Puskás, Judit Hohmann, István Zupkó

**Affiliations:** 1 Department of Pharmacodynamics and Biopharmacy, University of Szeged, H-6720 Szeged, Hungary; molnar.judit@pharm.u-szeged.hu; 2 AVIDIN Ltd., H-6726 Szeged, Hungary; g.szebeni@avidinbiotech.com (G.J.S.); laszlo@avidinbiotech.com (L.G.P.); 3 Department of Pharmacognosy, University of Szeged, H-6720 Szeged, Hungary; csuporboglar@gmail.com (B.C.-L.); hajdu@pharm.u-szeged.hu (Z.H.); hohmann@pharm.u-szeged.hu (J.H.); 4 Department of Medical and Chemical Laboratory Diagnostics, Medical University of Vienna, A-1090 Vienna, Austria; thomas.szekeres@meduniwien.ac.at (T.S.); philipp.saiko@meduniwien.ac.at (P.S.); 5 Department of Biochemistry, University of Szeged, H-6720 Szeged, Hungary; ocsovszki.imre@med.u-szeged.hu

**Keywords:** sesquiterpenes, leukemia cells, cell cycle, apoptosis

## Abstract

Plants and plant extracts play a crucial role in the research into novel antineoplastic agents. Four sesquiterpene lactones, artecanin (**1**), 3β-chloro-4α,10α-dihydroxy-1α,2α-epoxy-5α,7α*H*-guaia-11(13)-*en*-12,6α-olide (**2**), *iso*-*seco*-tanapartholide 3-*O*-methyl ether (**3**) and 4β,15-dihydro-3-dehydrozaluzanin C (**4**), were isolated from two traditionally used Asteraceae species (*Onopordum acanthium* and *Artemisia asiatica*). When tested for antiproliferative action on HL-60 leukemia cells, these compounds exhibited reasonable IC_50_ values in the range 3.6–13.5 μM. Treatment with the tested compounds resulted in a cell cycle disturbance characterized by increases in the G1 and G2/M populations, while there was a decrease in the S phase. Additionally, **1**–**3** elicited increases in the hypodiploid (subG1) population. The compounds elicited concentration-dependent chromatin condensation and disruption of the membrane integrity, as revealed by Hoechst 33258–propidium staining. Treatment for 24 h resulted in significant increases in activity of caspases-3 and -9, indicating that the tested sesquiterpenes induced the mitochondrial pathway of apoptosis. The proapoptotic properties of the sesquiterpene lactones were additionally demonstrated withannexin V staining. Compounds **1** and **2** increased the Bax/Bcl-2 expression and decreased the expressions of CDK1 and cyclin B2, as determined at the mRNA level by means of RT-PCR. These experimental results indicate that sesquiterpene lactones may be regarded as potential starting structures for the development of novel anticancer agents.

## 1. Introduction

Cancerous disorders are the leading cause of death in economically developed areas of the world and the second cause of mortality in developing countries. In the developing world, the cancer burden is increasing as a result of multiple factors, including the adoption of a “westernized” lifestyle. In 2008, there were 12.4 million newly diagnosed cancers and 7.6 million cancer-related deaths worldwide, and it is estimated that these values will increase to 26.4 and 17 million, respectively, by 2030 [1]. Both the current state and the tendencies in the global cancer data clearly indicate that the preventive and therapeutic modalities utilized at present are suboptimal, and novel modes of intervention are called for, including innovative drugs with improved efficacy profiles.

The plant kingdom is a well-established and abundant source of drug candidates, and the systematic screening of plant preparations is therefore a widely accepted approach for the identification of original compounds with promising pharmacological properties. A number of the currently used anticancer drugs (e.g., Vinca alkaloids, podophyllotoxin analogs or taxanes) were discovered in ethnomedicinally fortified screening programs of higher plants [2]. The initially identified natural product frequently exhibits auspicious activity, but with poor pharmacokinetic properties, and chemical derivatization is needed to obtain novel analogs suitable for further drug development. A recent comprehensive analysis revealed that only 6% of the currently used drugs are unmodified natural products, whereas 27% of them are closely related to a natural prototype and a further 30% were designed on the basis of a natural pharmacophore element. Approximately two-thirds of the present therapeutic armamentarium is therefore naturally derived or inspired, and this is especially true in regard to concerns anticancer agents [3].

**Figure 1:**
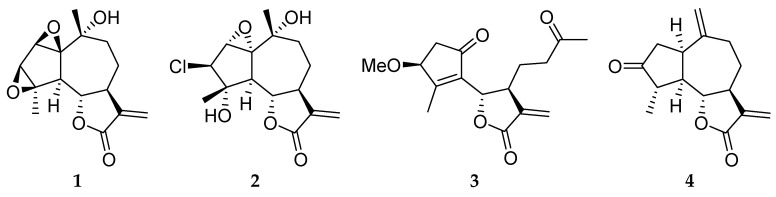
Chemical structures of the investigated sesquiterpenes.

It is estimated that the plant kingdom numbers around 250,000 species, of which approximately 6% have been studied for biological activity, and about 15% phytochemically. The Asteraceae comprise one of the largest families of higher plants, with over 13,000 species worldwide [4]. From a phytochemical aspect, the members of the family are outstandingly rich in secondary metabolites, including sesquiterpene lactones, flavonoids, polyacetylenes, lignans and pentacyclic triterpene alcohols [5]. Since many of the plants of the family are used ethnomedicinally for a wide array of indications, including malignant disorders [6], but only limited data are available concerning the antiproliferative properties of the European species, a broad systematic screening program was earlier initiated by our group [7,8]. More than 400 extracts were prepared from 51 Asteraceae species found in Central and Eastern Europe and were tested for antiproliferative and cytostatic effects on human adherent cancer cell lines. As a continuation of this work, several of the active extracts were subjected to activity-guided fractionation and isolation of the constituents responsible for the detected antiproliferative action. This resulted in new natural products and also known substances described first from the investigated plant [9,10]. One of the recently investigated plants was *Artemisia asiatica*, which has been utilized in traditional Asian medicine for many diseases, including cancers. A formulated extract is available in South Korea for the treatment of gastroduodenal injuries, but its cancer-preventive activity has also been demonstrated in animal tumor models [11,12]. From an extract of the *A. asiatica* herb, two guaianolides, artecanin (**1**) and 3β-chloro-4α,10α-dihydroxy-1α,2α-epoxy-5α,7α*H*-guaia-11(13)-*en*-12,6α-olide (**2**), and the seco-guaianolide *iso*-*seco*-tanapartholide 3-*O*-methyl ether (**3**) were recently isolated and demonstrated to exert antiproliferative actions against human adherent cancer cell lines [13]. *Onopordum acanthium* is another medicinal plant belonging in the Asteraceae family which has documented anticancer properties, and from this a fourth sesquiterpene lactone, 4β,15-dihydro-3-dehydrozaluzanin C (**4**), was isolated and tested in similar methods [14] (Figure 1).

The aim of our current investigations was to extend the investigations of these sesquiterpenes by testing their activities on the growth of HL-60 human promyelocytic leukemia cells. The most potent agents were subjected to additional *in vitro* experiments in order to elucidate their mechanisms of action. A set of sesquiterpene lactones were earlier found to be more effective on HL-60 leukemia cells than against a panel of adherent cells [15].

## 2. Results and Discussion

### 2.1. Antiproliferative Assay

**Figure 2:**
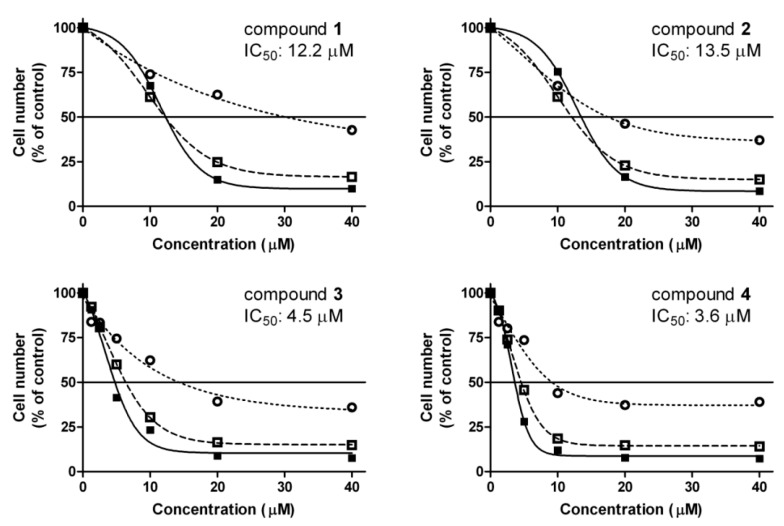
Concentration–response curves of **1**–**4** after incubation for 24 (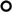), 48 (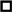) and 72 h (■) and their IC_50_ values calculated from 72-h data. Data are means ± SEM from three experiments.

The antiproliferative effects of the tested sesquiterpenes were determined by counting HL-60 cells after 24, 48 and 72 h of exposure, and the 72-h results were used to calculate IC_50_ values (Figure 2). Compounds **3** and **4** proved to be more potent than **1** and **2**, which is in good agreement with our previous data on adherent human cancer cell lines [13,14].

### 2.2. Cell Viability Measurements

**Figure 3:**
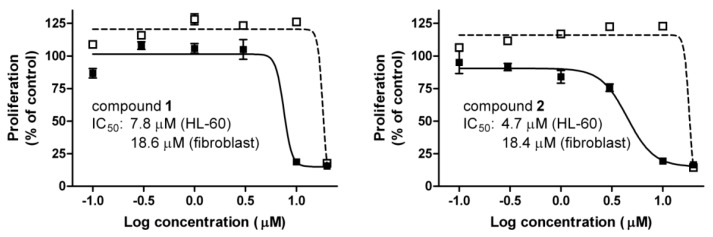
Concentration–response curves of **1** and **2** after incubation for 72 h, and their calculated IC_50_ values. 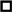 and ■ indicate fibroblast and HL-60 cells, respectively. Data are means ± SEM from three experiments.

The cancer selectivities of **1** and **2** were characterized by means of MTS viability assays after 72 h of incubation against HL-60 and fibroblasts (Figure 3). Both of the tested sesquiterpenes exerted substantially more pronounced antiproliferative action against HL-60 than fibroblasts, indicating selective inhibition of cancer cell proliferation.

### 2.3. Cell Cycle Analysis

The effects of the tested compounds on the cell cycle distribution were determined by means of flow cytometry. HL-60 cells were treated with the compounds at 5 and 10 μM for 24 and 48 h and the cell populations in the various cell cycle phases (subG1, G1, S and G2/M) were measured.

**Figure 4:**
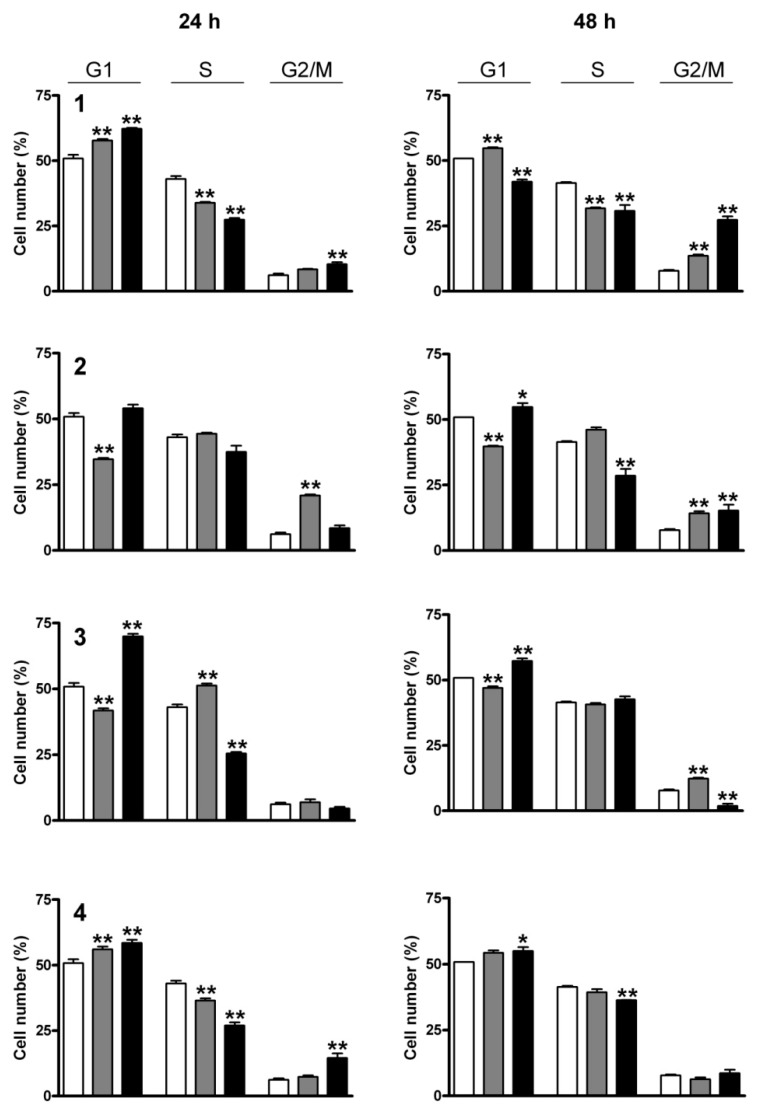
Cell-cycle distributions of HL-60 cells after treatment with **1**–**4** for 24 and 48 h. Open, gray and black columns indicate data from control cells and cells treated with 5 or 10 µM test substance, respectively. * and ** denote *p* < 0.05 and *p* < 0.01, respectively, as compared with the control condition. Data are means ± SEM from three determinations.

**Figure 5:**
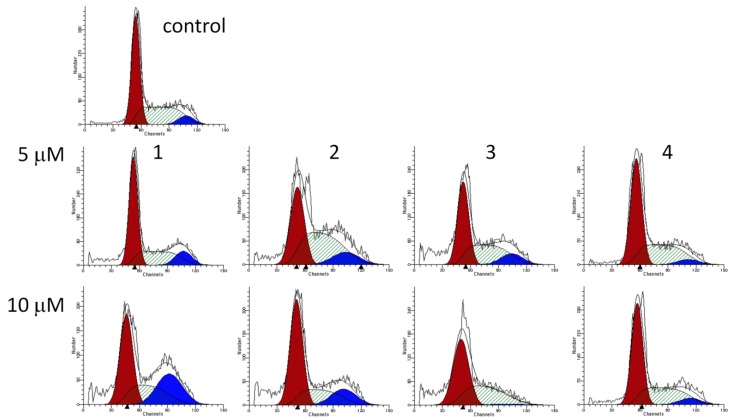
Representative cell-cycle histograms of control HL-60 cells after treatment with 5 or 10 µM **1**–**4** for 48 h.

**Figure 6:**
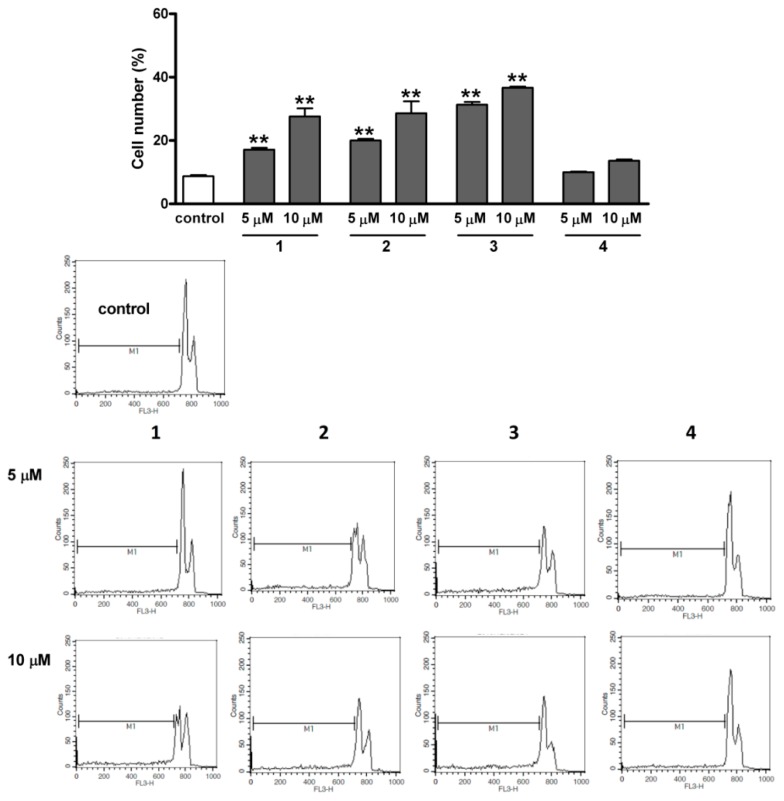
Hypodiploid (subG1) population of HL-60 cells after treatment with **1**–**4** for 48 h (**upper panel**). ** denotes *p* < 0.01 as compared with the control condition. Data are means ± SEM from three determinations. Histograms representing the same conditions (**lower panel**).

Each of the four sesquiterpenes caused a concentration-dependent disturbance of the cell cycle distribution (Figure 4 and Figure 5). In general, the most pronounced results of treatment were increases in the G1 and G2/M populations, while the number of cells in the S phase was decreased. The action on the G2/M population was typically more substantial after 48 h than after 24 h of incubation, which could indicate a cell cycle arrest at the G2/M phase. The hypodiploid (subG1) population was additionally determined and a concentration and exposure-dependent increase was observed after 48 h of incubation. This latter action was a pronounced exception for **4** (Figure 6). Shorter (24 h) exposure did not result in a substantial increase in subG1 cells (data not shown).

### 2.4. Fluorescent Staining

**Figure 7:**
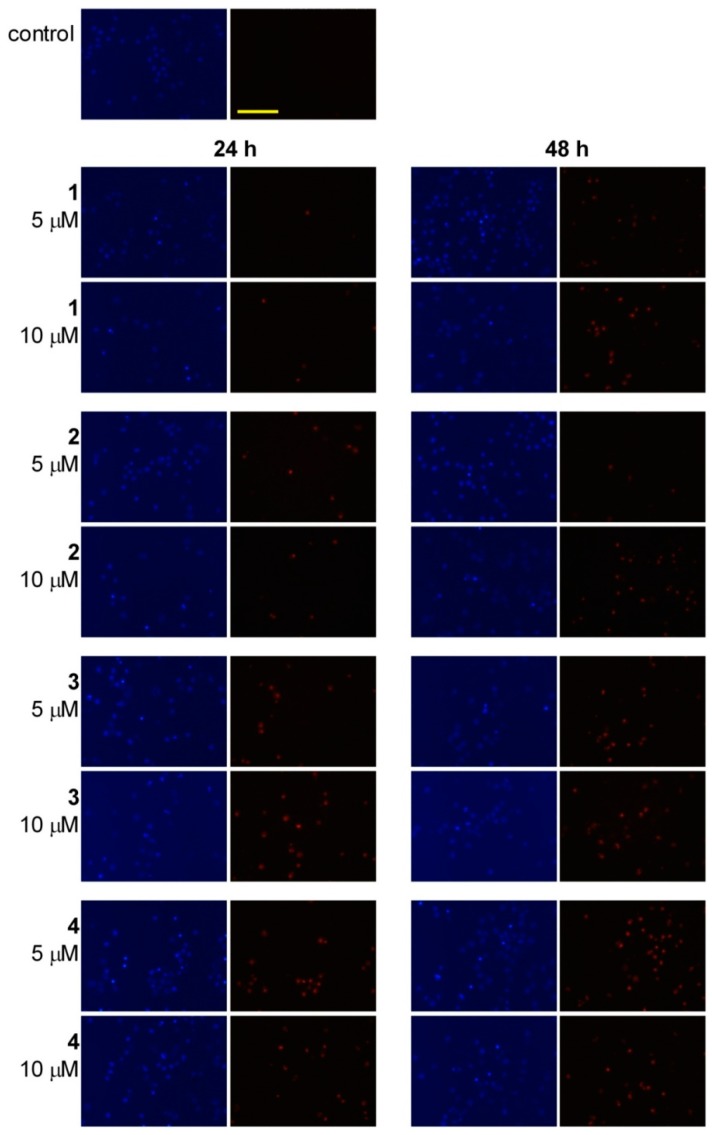
Fluorescence microscopic images of sesquiterpenes **1**–**4** after incubation for 24 and 48 h. Two separate pictures from the same field were taken for the two markers. The blue fluorescence (**left panels**) relates to Hoechst 33258, and the red coloration (**right panels**) reflects cellular PI accumulation. The bar in the PI control picture indicates 100 µm.

HL-60 cells were incubated with 5 and 10 μM sesquiterpenes for 24 and 48 h and double-stained with fluorescent DNA markers (Hoechst 33258 and propidium iodide (PI)) to evaluate the morphological consequences. More intensive staining with Hoechst 33258 can be interpreted as a consequence of nuclear condensation. Separate pictures were taken illustrating the Hoechst 33258 and PI fluorescence as morphological markers. The extents of nuclear fragmentation and condensation were concentration-dependent for all four natural products after both incubation times, but were relatively independent of time after 24 h (Figure 7). Treatment-related impairment of the membrane function was detected using more intense PI staining, which was concentration- and exposure-dependent. Treatment with **3** or **4** resulted in profoundly disturbed membrane permeability, indicating a higher contribution of necrosis induction.

### 2.5. Determination of Caspase-3 and Caspase-9 Activities

Most of the current drugs applied in tumor therapy have the capacity to initiate apoptotic cell death, and the resultant controlled self-disassembly of the cancer cell is more advantageous to the surrounding intact cells than if necrosis occurs. A potential anticancer lead compound is expected to induce programmed cell death by modifying the balance between apoptotic and antiapoptotic signaling. Demonstration of a proapoptotic property is therefore a crucial part of the investigation of a novel potential antiproliferative drug candidate [16]. Although an increase in the subG1 population is also a generally accepted marker of apoptosis, one of the most reliable indicators of this is activation of the specific enzymes involved in the controlled cellular decomposition.

Caspases are cysteinyl aspartate proteases with elementary functions in the apoptotic machinery. The 18 mammalian caspases are classified as initiator or executioner caspases according to their role in the procedure [17].

**Figure 8:**
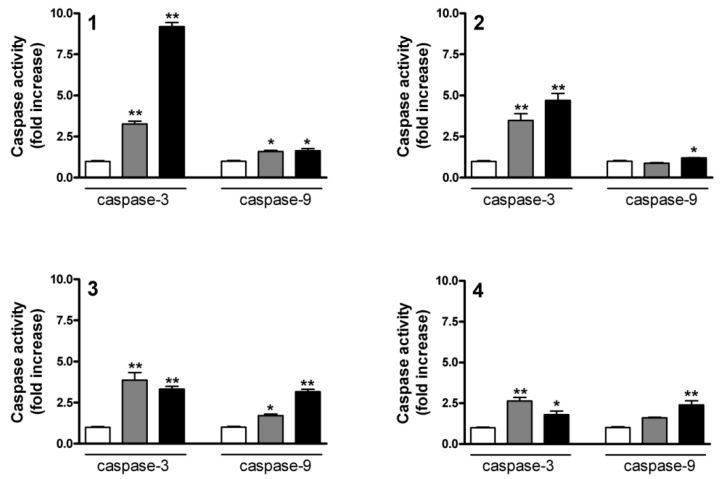
Induction of caspase-3 and caspase-9 activities after incubation with compounds **1**–**4** for 24 h. White, gray and black columns denote the control, or 5 and 10 µM sesquiterpene, respectively. * and ** denote *p* < 0.05 and *p* < 0.01, respectively, as compared with the control condition. The activities of caspase-3 and caspase-9 were determined with fluorimetric and colorimetric assay kits, respectively. Data are means ± SEM from three determinations.

Since no substantial incubation time-dependent differences were revealed by cell cycle analysis and fluorescence microscopy after 24 h, the activities of the crucial caspases were determined only after a 24-h exposure. All four sesquiterpenes resulted in a significant increase in the activity of the main executionary isoenzyme caspase-3, the effects of **1** and **2** proving more pronounced (Figure 7). In the cases of **3** and **4**, the action at 10 µM was less marked than that at 5 µM. **1**–**4** all increased the activity of caspase-9, the initiator enzyme of the intrinsic pathway of apoptosis [17], though less extensively than in the case of caspase-3 (Figure 8). Caspase-9 is the first element in a cascade, whereas caspase-3 is a terminal element, *i.e.*, a product of amplification.

### 2.6. Flow Cytometric Apoptosis Assay

**Figure 9:**
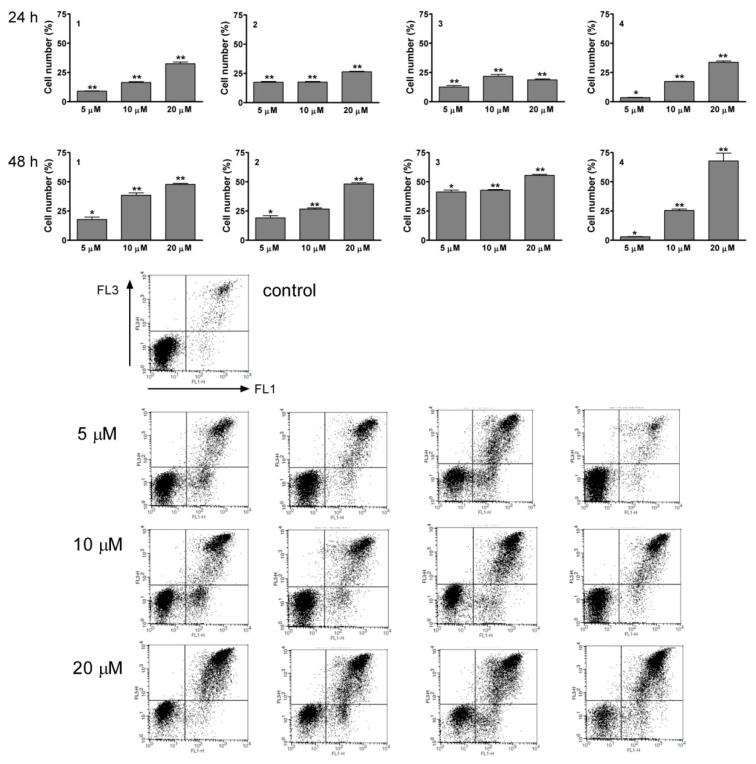
Proportion ofannexin V positivity after incubation with compounds **1**–**4** for 24 and 48 h (**upper panels**). * and ** denote *p* < 0.05 and *p* < 0.01, respectively, as compared with the control condition. Baseline values have been subtracted from treated values. Data are means ± SEM from three determinations. Representative dot-plots from 48-h treatments (**lower panels**).

The proapoptotic potentials of **1**–**4** were further investigated by means of annexin V-Alexa 488 and PI staining, followed by flow cytometry. Treatment with each of the natural products resulted in concentration- and time-dependent increases in annexin V-positive cells (Figure 9). **1**–**4** elicited significant increases after 24 h of treatment, even at the lowest concentration used (5 µM). Annexin V positivity indicates the loss of physiological phospholipid asymmetry maintained by the enzyme aminophospholipid translocase. During the early stages of programmed cell death, phosphatidylserine is exposed to the outer surface of the cell membrane and this phenomenon is considered to be a biochemical apoptotic marker [18].

### 2.7. RT-PCR Studies

The mitochondria can be considered the central hub in the regulation of the apoptosis–survival balance. The intrinsic pathway of apoptosis is triggered by the release of cytochrome c and the subsequent recruitment of executionary proteins. This initiating function of the mitochondria is governed by the members of the Bcl-2 protein family, including antiapoptotic factors Bcl-2, Bcl-xl and Mcl-1 and the proapoptotic members Bax and Bak [19]. Bax and Bcl-2 are the most frequently investigated members of the family and their ratio is regarded as a descriptor of the actual apoptotic–survival state.

**Figure 10:**
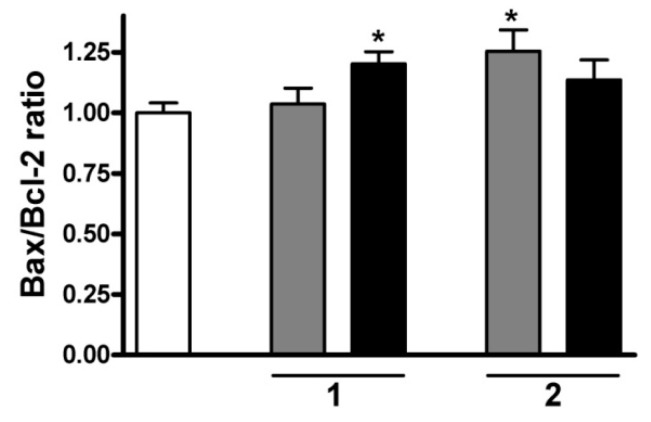
Calculated ratios Bax/Bcl-2 at an mRNA level after incubation with compound **1** or **2** for 24 h. White, gray and black columns relate to the control, or 5 and 10 µM sesquiterpene, respectively. * denotes *p* < 0.05, as compared with the control condition. Data are means ± SEM from three determinations.

Sesquiterpenes **1** and **2** were selected for determination of the mRNA-level expression of these factors. A 24-h treatment with **1** resulted in an increase in the calculated ratio Bax/Bcl-2, which was found to be significant at 10 µM, while **2** elevated this ratio at 5 µM, but not at 10 µM (Figure 10). Compounds **1** and **2** exhibited very similar IC_50_ values. Although the treatment-related activation of both caspases was less pronounced for **2** and the higher concentration elicited only a modest and nonsignificant change in the ratio Bax/Bcl-2, it could be concluded that this agent may induce non-apoptotic, caspase-independent cell death, especially at higher concentrations.

**Figure 11:**
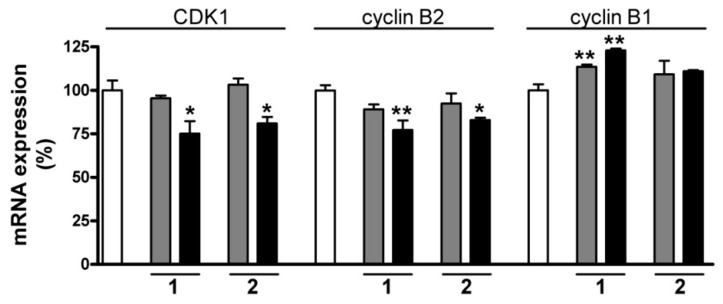
Expression of CDK1, cyclin B1 and cyclin B2 at the mRNA level after incubation with **1** or **2** for 24 h. White, gray and black columns relate to the control, or 5 and 10 µM sesquiterpene, respectively. * and ** denote *p* < 0.05 and *p* < 0.01, respectively, as compared with the control condition. Data are means ± SEM from three determinations.

Since the cell cycle analysis results clearly indicate an arrest at the G2/M phase, the regulatory background of this blockade was additionally investigated. The multiplication of the cells is a highly complex procedure, involving a not yet completely described array of regulatory circuits. As concerns the G2/M phase transition, the complex formed by cyclin B1, B2 and cyclin-dependent kinase 1 (CDK1) plays a crucial role [20]. The mRNA-level expressions of these three factors were additionally determined after treatment for 24 h with **1** or **2**. Both sesquiterpenes at 10 µM decreased the expression of CDK1 and cyclin B2. The expression of cyclin B1 was increased by **1** while no considerable changes were detected after treatment with **2** (Figure 11). Many cell-cycle-governing proteins, including these two factors, are regulated by postsynthetic phosphorylation, and determination of the mRNA-level expression is therefore not the optimal approach for an exact description of their function. However, the effects of the sesquiterpenes on these factors may indicate that these natural products exert their antiproliferative action by disturbing the cell cycle machinery.

The plant kingdom contains a virtually inexhaustible source of natural products with attractive pharmacological properties, which makes plant extracts suitable subjects for lead-finding studies. The predominance of drugs that have been developed from or inspired by natural products is especially true for anticancer agents. Such effective plant preparations may serve not only as the source of the active agent, but additionally as a safe and cost-effective means of chemoprevention [21]. The sesquiterpene lactones constitute a chemically diverse group of more than 5000 secondary metabolites, most of them isolated from Asteraceaea species. These natural products possess diverse biological activities, their anti-inflammatory and antiproliferative properties being the most characteristic [22]. Guaianolides and pseudoguaianolides are the subclasses of sesquiterpenes that exhibit the most pronounced cytotoxic effects, which are attributed to two main structural components: α-methylene-γ-lactone and α,β-unsaturated cyclopentanone rings [23]. These structural moieties, which function as alkylating agents reacting with sulfhydryl groups, are needed for antiproliferative action. This Michael-type addition involves various intracellular targets including mercaptyl groups of proteins, leading to the loss of enzyme activity [24]. The reaction also results in the depletion of free intracellular glutathione, disruption of the cellular redox balance and the consequent oxidative stress [25]. The generated reactive oxygen species have the capacity to activate the mitochondrial pathway of apoptosis [26]. Sesquiterpenes containing an epoxide ring besides the α-methylene-γ-lactone pharmacophore can be regarded as bifunctional molecules and more pronounced antiproliferative action can be expected from them [24] Two of the currently investigated natural products,**1** and **2**, are such sesquiterpenes. The consequences of the alkylation have been only incompletely elucidated, but one of the best-characterized sesquiterpenes with an anticancer effect is parthenolide, which directly inhibits NF-κB and also prevents the phosphorylation and subsequent degradation of IκB [27,28].

The terpenes are a “drug-like” subclass of natural compounds. Ingenol mebutate, found in *Euphorbia peplus*, has been registered for the treatment of premalignant lesions in actinic keratosis [29]. Orally active parthenolide derivatives have been prepared and proved selective against cancer stem cells [28]. The water-soluble dimethylaminohydrochloride derivative of the sesquiterpene arglabin is used in oncological practice in Kazakhstan [30]. All of our currently investigated effective molecules contain these critical pharmacophores, as an indication that they may be regarded as potential starting structures for the design and development of novel anticancer agents.

## 3. Experimental Section

### 3.1. Chemicals

The currently investigated sesquiterpene lactones **1**–**4** were isolated and identified as described earlier [13,14]. Their chemical structures are presented in Figure 1. All other chemicals, if otherwise not specified, were from Sigma–Aldrich (Budapest, Hungary).

### 3.2. Cell Culturing and Antiproliferative Assay

The human HL-60 promyelocytic leukemia cell line was purchased from the American Type Culture Collection (Manassas, VA, USA). Cells were grown in RPMI 1640 medium supplemented with 10% heat inactivated fetal calf serum (FCS), 1% l-glutamine, and 1% penicillin-streptomycin at 37 °C in a humidified atmosphere containing 5% CO_2_, using a Heraeus cytoperm 2 incubator (Heraeus, Vienna, Austria). Human skin fibroblasts (Sigma–Aldrich, Budapest, Hungary) were maintained in DMEM/F12 medium supplemented with 10% FCS. All media and supplements were purchased from Gibco Life Technologies (Paisley, Scotland, UK). Cell counts were determined with a CC-110 microcellcounter (Sysmex, Kobe, Japan). Cells growing in the logarithmic phase of growth were used for all experiments described below.

The antiproliferative effects of **1**–**4** on HL-60 cells were determined by cell counting. Briefly, cells were seeded in 25-cm^2^ tissue culture flasks at a density of 750,000 cells/5 mL medium and the cells were exposed to the tested compound. After incubation periods of 24, 48 and 72 h, the cells were counted with a cell counter. All *in vitro* experiments were carried out on at least three parallel samples. Sigmoidal dose–response curves were fitted to the measured points and IC_50_ values were calculated by means of GraphPad Prism 4 software (GraphPad Software, San Diego, CA, USA).

### 3.3. Cell Viability Measurements

The viability of HL-60 human leukemia cells and fibroblasts from healthy donors was determined by the colorimetric MTS assay (Promega, Madison, WI, USA) as described earlier [31]. Briefly, the cells (4000 HL-60 and 6000 fibroblasts) were seeded into 96-well plates in RPMI 10% FCS and DMEM/F12 10% FCS, respectively. Cells were cultured overnight before treatment. The effects of **1** and **2** at 0, 0.1, 0.3, 1, 3, 10 and 20 µM were examined after a 72-h incubation. The MTS reagent (3-(4,5-dimethylthiazol-2-yl)-5-(3-carboxymethoxy-phenyl)-2-(4-sulfophenyl)-2*H*-tetrazolium) was applied to drug-treated and control (0.2% DMSO) cells according to the manufacturer’s protocol. Absorbance (at 490 nm) was recorded on a multimode microplate reader (Perkin Elmer, Waltham, MA, USA). Viability was calculated relative to untreated control cells and blank wells containing media without cells.

### 3.4. Cell Cycle Analysis by Flow Cytometry

Cellular DNA content was measured by means of flow cytometric analysis, using the DNA-specific fluorescent dye PI. HL-60 cells (200,000) were plated in 24-well tissue culture plates (Corning Life Sciences, Corning, NY, USA) in RPMI 10% FCS and were treated with the indicated compound and concentrations on the graphs. After 24 or 48 h the cells were collected, washed with PBS and resuspended in DNA binding buffer (1× PBS, 0.1% trisodium citrate, 10 µg/mL PI, 0.1% Tx, 10 µg/mL RNaseA, (Sigma–Aldrich)). After 30 min incubation at room temperature cells were acquired on a FACSCalibur cytofluorimeter (Becton Dickinson, Franklin Lakes, NJ, USA), we gated out doublets for cell cycle analysis which was based on FL2-A/FL2-W dot plots, using ModFit software (Verity Software House, Topsham, ME, USA). Sub-G1 apoptotic population was analyzed on FL3 histograms using CellQuest software (Becton Dickinson) [32].

### 3.5. Double Staining with Hoechst 33258 and PI

Cells were seeded into a 6-well plate and incubated with various concentrations of the tested compounds for 24 or 48 h. Hoechst 33258 and PI were added directly to the cells to give final concentrations of 5 and 3 µg/mL, respectively. After incubation for 60 min at 37 °C, the cells were examined on a Nikon Fluorescence Microscope equipped with a Digital Sight Camera System, including appropriate filters for Hoechst 33258 and PI [33,34].

### 3.6. Caspase-3 Assay

The activity of caspase-3 from treated cells was determined by means of a fluorimetric assay kit (Sigma–Aldrich Ltd., Budapest, Hungary). Ac-DEVD-AMC was used as substrate. In the course of the assay, the peptide substrate was cleaved by the enzyme, resulting in the release of AMC, which was determined on a microplate reader at 360/460 nm (excitation/emission). HL-60 cells were treated with the tested compounds at 5 and 10 µM for 24 h, untreated cells serving as control. The cells were harvested and incubated with cell lysis buffer (in proportion to the cell number) on ice for 15 min. The cell lysate was centrifuged (15 min, 17,000× *g*) and the supernatant was collected and analyzed. The results were expressed in fold increase of caspase-3 activity relative to the result of the control condition [35].

### 3.7. Caspase-9 Assay

Caspase-9 activity was determined with a colorimetric assay kit (Sigma–Aldrich Ltd., Budapest, Hungary). Ac-LEHD-*p*NA was used as substrate. The cleavage of the peptide substrate by caspase-9 resulted in the release of *p*NA, which was measured on a microplate reader at an absorbance wavelength of 405 nm. All further conditions were identical with those in the caspase-3 assay.

### 3.8. Reverse Transcription-Polymerase Chain Reaction (RT-PCR) Studies

The actions of the investigated sesquiterpenes on the mRNA expression pattern of the markers of apoptosis, such as Bax, Bcl-2, cyclin-dependent kinase 1 (CDK1), cyclin B1 and cyclin B2, which play fundamental roles in the G2–M transition, were determined by RT-PCR in HL-60 cells. After a 24-h incubation period, the medium containing the tested natural products was discarded and the total RNA was isolated from the cells (4 × 10^5^) by using the TRIzol Reagent according to the instructions of the manufacturer (Csertex Ltd., Budapest, Hungary). The pellet was resuspended in 100 μL DNase- and RNase-free distilled water. The concentrations of RNA in the samples were determined from their absorbances at 260 nm. The RNA (0.5 μg) was mixed with DNase- and RNase-free distilled water and 20 μM oligodT (Invitrogen, Carlsbad, CA, USA) in a final reaction volume of 10 μL, and incubation was performed at 70 °C for 5 min. After the mixture had been cooled to 4 °C, 20 U MMLV reverse transcriptase (Promega, Madison, WI, USA), 20 U RNase inhibitor (Promega, Madison, WI, USA), 200 μM dNTP (Sigma–Aldrich; Budapest, Hungary) in 50 mM Tris–HCl, pH 8.3, 75 mM KCl, and 5 mM MgCl_2_ in a final reaction volume of 10 μL were added. The mixture was incubated at 37 °C for 60 min. The reaction was performed in the presence of 5 μL cDNA, 12.5 μL GoTaq Green Master Mix, 2 μL 20 pM sense and antisense primers of Bax, Bcl-2, CDK1 and cyclin B2 and 3.5 μL DNase- and RNase-free distilled water. Human glyceraldehyde 3-phosphate dehydrogenase (hGAPDH) primers were used as internal control in all samples (Table 1). The reaction was performed with an ESCO SWIFT MAXI thermal cycler (Esco Technologies Inc., Philadelphia, PA, USA) and the products were separated on 2% agarose gels, marked with ethidium bromide and photographed under a UV transilluminator. Semiquantitative analysis was obtained by means of densitometric scanning of the gel with Kodak IMAGE STATION 2000R (Csertex Ltd., Budapest, Hungary) [36].

**Table 1:** Primers and PCR conditions of the determined genes, the Genebank access numbers and the length of PCR products.

Name	Primer Sequence	Gene ID	Product Size (bp)	Coupling Temp. (°C)
CDK1	F: ACTGGCTGATTTTGGCCTTGCC	983	118	62
	R: TGAGTAACGAGCTGACCCCAGCAA			
cyclin B1	F: AATAAGGAGGGAGCAGTGCG	891	51	60
	R: GAAGAGCCAGCCTAGCCTCAG			
cyclin B2	F: GCGTTGGCATTATGGATCG	9133	51	60
	R: TCTTCCGGGAAACTGGCTG			
Bax	F: TGGCAGCTGACATGTTTTCTGAC	581	195	53
	R: CGTCCCAACCACCCTGGTCT			
Bcl-2	F: GACTTCGCCGAGATGTCCAG	596	225	51
	R: CAGGTGCCGGTTCAGGTACT			
hGAPDH	F: ACCCAGAAGACTGTGGATGG	2597	415	55
	R: TGCTGTAGCCAAATTCGTTG			

### 3.9. Flow Cytometric Apoptosis Assay

HL-60 cells (200,000) were plated in 24-well tissue culture plates in RPMI medium supplemented with 10% FCS and were treated with **1**–**4** in the concentrations indicated in the graphs. After 24 or 48 h, the cells were collected and resuspended in Annexin V binding buffer (0.01 M HEPES, 0.14 M NaCl and 2.5 mM CaCl_2_). Annexin V-Alexa 488 (LifeTechnologies, Waltham, MA, USA) was added to the cells, which were then kept in the dark for 15 min at room temperature. Before the acquisition, PI (10 μg/mL) in annexin V binding buffer was added to diluted annexin V-Alexa 488 5-fold. Cells were analyzed on a FACSCalibur cytofluorimeter, using CellQuest software (Becton Dickinson, Franklin Lakes, NJ, USA). The percentages of the FL1 (annexin V-Alexa 488) positive and FL3 (PI) negative early-apoptotic cells and FL1 (annexin V-Alexa 488) positive and FL3 (PI) positive late-apoptotic cells were determined [37].

### 3.10. Statistical Analysis

All *in vitro* experiments were performed in triplicate and statistical analysis was carried out by ANOVA, followed by the Dunnett post-test with the use of GraphPad Prism 4 software (GraphPad Software, San Diego, CA, USA).

## 4. Conclusions

Overall, the presented results indicate that sesquiterpenes **1**–**4** exert antiproliferative action against HL-60 human leukemia cells. Although the sesquiterpenes are widely investigated compounds and their anticancer action is well recognized, similar results have not been published previously concerning **1**–**4**. It was demonstrated that **1**–**4** elicited a concentration-dependent disturbance in the cell cycle and induced apoptosis by activating the mitochondrial pathway. The presented data indicate that these sesquiterpenes can be regarded as suitable structures for the development of novel potential anticancer agents.
